# Advances and Prospects in Odour-based Management of Filth Flies

**DOI:** 10.1007/s10886-026-01705-7

**Published:** 2026-04-22

**Authors:** Steve B. S. Baleba

**Affiliations:** 1 International Centre of Insect Physiology and Ecology, PO Box 30772-00100, Nairobi, Kenya; 2 Department of Zoology and Entomology, University of Pretoria, Private Bag X20, Hatfield, 0028 South Africa

**Keywords:** Semiochemicals, Insect olfaction, Attraction, Repellency, Trapping systems, Integrated pest management

## Abstract

Filth flies are groups of dipteran species closely associated with human and animal faeces, food waste, or carrion. They include species such as the house fly (*Musca domestica*), face fly (*Musca autumnalis*), bazaar fly (*Musca sorbens*), false stable flies (*Muscina stabulans*, *Muscina levida*), lesser house fly (*Fannia canicularis*), stable flies (*Stomoxys* spp.), horn fly (*Haematobia irritans*), neglected blood feeder fly (*Musca crassirostris*), oriental latrine fly (*Chrysomya megacephala*), green bottle fly (*Lucilia sericata*), Australian sheep blowfly (*Lucilia cuprina*) and blue bottle fly (*Calliphora vicina*). Thriving on decomposing organic waste, sewage, animal faeces, and carrion, these flies act as mechanical vectors of viruses, bacteria, protozoa, and helminths. They also cause direct harm through annoyance, stress, skin lesions, blood loss, and immunosuppression. In humans, contact with contaminated items, body parts, or food results in diseases such as trachoma, chlamydia, cholera, and gastrointestinal infections. In livestock, infestations lead to reduced weight gain and milk yield, causing economic losses exceeding USD 2 billion annually. Like all insects, filth flies rely heavily on olfaction to locate food, mates, oviposition sites, shelters, and to avoid danger. Identifying the odour cues that guide these behaviours offers promising opportunities for control, particularly when integrated into trapping or repellent systems. This review summarises current knowledge on filth fly management using odour cues. It explains the nature of these cues and their detection mechanisms in insects, discusses studies applying them for monitoring, attraction, repellency, or mass trapping, and highlights key considerations for developing effective odour-based control strategies. The review provides a useful reference for veterinarians, entomologists, chemical ecologists, industry, and funding agencies seeking sustainable alternatives for managing filth flies.

## Introduction

**Fig. 1:**
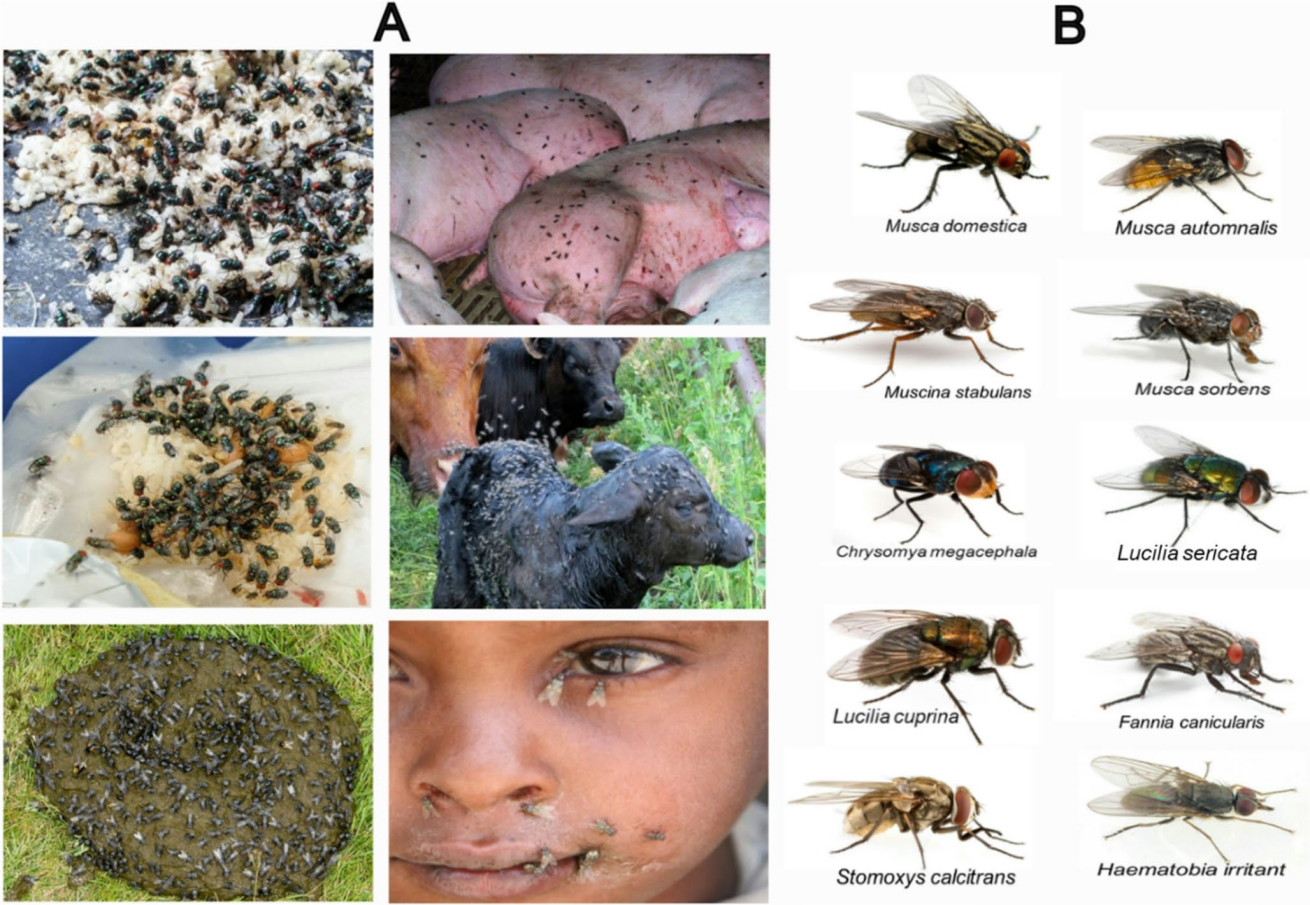
Filth fly species commonly found near human and animal habitats. **A** Food waste, human and animal faeces, a pig, a calf, and a child, all visibly covered with filth flies. **B** Representative fly species associated with these environments. Photo sources: https://www.greenleafpestcontrol.com; https://www.istockphoto.com/, https://ganadosycarnes.com/; https://www.ces.ncsu.edu/; https://www.sciencephoto.com/; https://www.pond5.com/stock-fo

When observing areas with garbage and sewage, or visiting farms with cattle, sheep, horses, pigs, or poultry, it is common to see many colourful insects (Fig. 1A). These are often filth flies closely associated with humans and animals. They include species such as the house fly (*Musca domestica*), face fly (*Musca autumnalis*), bazaar fly (*Musca sorbens*), false stable flies (*Muscina stabulans*, *Muscina levida*), lesser house fly (*Fannia canicularis*), stable flies (*Stomoxys spp.*), horn fly (*Haematobia irritans*), neglected blood feeder fly (*Musca crassirostris*), oriental latrine fly (*Chrysomya megacephala*), green bottle fly (*Lucilia sericata*), sheep blowfly (*Lucilia cuprina*) and blue bottle fly (*Calliphora vicina*) (Fig. 1B). These flies use faeces, food waste, or carcasses for the development of their immature stages (eggs, larvae, and pupae). Depending on their mouthparts at the adult stage, they either feed on body secretions or act as blood-feeding ectoparasites. House flies (Geden et al. 2021), face flies (Broce and Elzinga 1984), bazaar flies, and lesser house flies (Moon 2019) possess a spongy proboscis with prestomal teeth incapable of piercing the skin. They are facultative blood feeders, relying on organic wastes, excrements, carcasses, lacrimal fluids, and secretions from wounds caused by biting flies. In contrast, stable flies (Cilek 2004), horn flies (Fernandes et al. 2020), and neglected blood feeder flies (Desquesnes et al. 2019) are true biting species. Both males and females are haematophagous and possess a strongly chitinized proboscis that pierces the host’s skin to draw blood. Each fly can take about 15 µL of blood per meal (Foil and Hogsette 1994). Since large numbers attack repeatedly, the cumulative blood loss can have serious veterinary implications.

During peak infestations, filth flies cause both direct and indirect damage to humans and animals. Direct effects include skin lesions, reduced food intake, stress, and blood loss. Indirectly, they transmit numerous pathogens, including viruses, bacteria, protozoa, nematodes, and helminths. Several species are linked to human diseases. *Musca sorbens* transmits *Chlamydia trachomatis*, the causative agent of trachoma, a neglected tropical disease affecting over 1.8 million people in sub-Saharan Africa (Emerson et al. 2000; Ramesh et al. 2016). *Chrysomya megacephala*, *Lucilia cuprina*, *Lucilia sericata* and *Calliphora vicina* act as mechanical carriers of enteric bacteria such as *Salmonella spp.*, *Escherichia coli*, *Shigella spp.*, and *Vibrio spp.* (Chaiwong et al. 2014; Tomberlin et al. 2017; Junqueira et al. 2017). *C. megacephala* also carries helminths and protozoa, including *Ascaris spp.*, *Trichuris spp.*, *Giardia spp.*, *Cryptosporidium spp.*, *Taenia spp.*, *Hymenolepis spp.*, *Entamoeba spp.*, *Toxocara spp.*, and *Toxoplasma gondii* (Patel et al. 2022; Zhao et al. 2014). Although evidence for *L. cuprina* and *C. vicina* is limited, both have been found to harbour enteric bacteria and parasite cysts, suggesting similar potential (Dar and Mir 2024). In a systematic review, Khamesipour et al. (2018) reported over 130 pathogens, mostly bacteria, carried by *Musca domestica*, highlighting their importance in human disease transmission. According to the World Health Organisation, infections by these pathogens cause about 2.2 million deaths annually, mostly among children in Africa and Southeast Asia (Mafokwane et al. 2023).

Filth flies also transmit animal pathogens. *Musca autumnalis* spreads *Moraxella* and *Brucella* bacteria, which cause bovine keratoconjunctivitis and brucellosis (Trout Fryxell et al. 2021). They also transmit *Thelazia* nematodes that infect mammalian eyes (Otranto and Traversa 2005) and *Parafilaria bovicola*, which produces subcutaneous lesions on cattle (Hund et al. 2021). *Haematobia irritans* spreads *Staphylococcus aureus* among dairy herds (Ryman et al. 2013). *Stomoxys calcitrans* transmits Rift Valley fever virus (Hoch et al. 1985), *Bacillus anthracis* (Hugh-Jones and Blackburn 2009; Turell et al. 1987), *Trypanosoma evansi* (Sumba et al. 1998), and *Habronema microstoma* (Traversa et al. 2008). *Fannia canicularis* transmits Newcastle disease virus (Chakrabarti et al. 2007), Aleutian mink disease virus (Prieto et al. 2018), and bacteria such as *Campylobacter spp.* (Royden et al. 2016), *Bacillus subtilis*, *Enterococcus spp.*, *Staphylococcus aureus*, and *Pantoea spp.* (Boiocchi et al. 2019). Economic losses caused by these infestations are significant. In the United States, cattle producers lose nearly one billion dollars annually due to horn fly and house fly infestations (Cupp et al. 1998; Geden et al. 2021). Stable flies alone cause losses estimated at $2.2 billion per year (Taylor et al. 2012). In Sweden, *P. bovicola* transmitted by face flies resulted in losses of about $8 million (Bech-Nielsen et al. 1983).

Over the last three decades, extensive research has generated new insights into filth fly control. Approaches include sanitation, mass trapping, physical barriers, biological control agents (predators, parasites, fungi, microsporidia, nematodes, and bacterial endosymbionts), insecticides (e.g., cyfluthrin, cyhalothrin, cypermethrin, dichlorvos, diflubenzuron), and semiochemical cues (pheromones, kairomones, allomones, and synomones). This review does not aim to cover all these strategies. Instead, it focuses on studies that isolate and identify semiochemical cues that mediate key behaviours in filth flies, such as feeding, mating, oviposition, aggregation and avoidance, and explores how these cues can be exploited for their control. First, I define semiochemical cues and explain how insects detect them. Then, I discuss studies applying these cues as control tools. Finally, I outline key factors to consider when designing management strategies based on semiochemicals. I hope this review will encourage insect chemical ecologists and neuroethologists to expand their research to include filth flies, beyond the traditional focus on mosquitoes, sandflies, tabanids, and tsetse flies.

## Semiochemical Cues: Definition and Detection in Insects

### Definitions

**Fig. 2:**
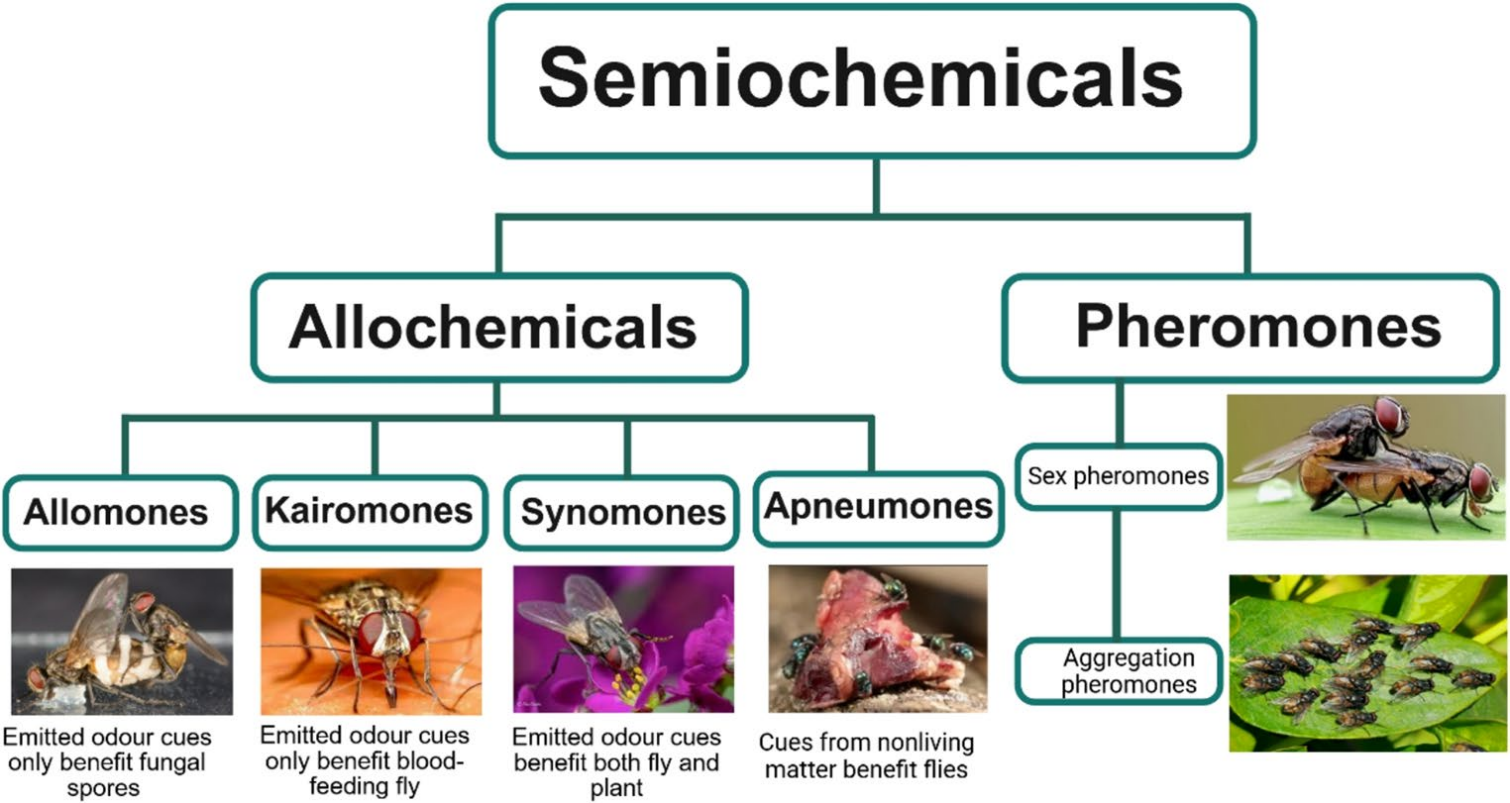
Schematic representation of semiochemical classes divided into pheromones and allelochemicals (allomones, kairomones, synomones, and apneumones), with examples illustrating their ecological functions in intra- and interspecific communication. Photo sources: Naundrup et al. (2022), Nitin Chandra; https://www.gingin.wa.gov.au/stable-fly-management; https://www.shutterstock.com/; https://www.discoverwildlife.com; https://pixabay.com/

Semiochemicals are molecular signals that mediate communication within and between species of animals and plants (Regnier 1971). They include various chemical classes such as acids, alcohols, aldehydes, amines, aromatics, esters, ketones, lactones, sulfur compounds, and terpenes. Nordlund and Lewis (1976) classified semiochemicals into pheromones and allelochemicals, depending on whether communication occurs within or between species (Fig. 2). Pheromones are low-molecular-weight compounds that elicit behavioural responses among individuals of the same species and facilitate intraspecific communication between males and females (Fleischer and Krieger 2018). They include sex pheromones (which attract potential mates over long distances) and aggregation pheromones (promoting group formation and mating). Other categories include alarm pheromones (released during predator attack), anti-aggregation pheromones (maintaining spacing under resource limitation), oviposition-deterring pheromones (reducing intraspecific competition at oviposition sites), and trail pheromones (guiding colony members) (Tewari et al. 2014; Yew and Chung 2015). In contrast, allelochemicals are chemicals produced by one organism that influence the behaviour, growth, or survival of another species (Mbaluto et al. 2020; Tlak Gajger and Dar 2021). Based on the benefit to the emitter and/or receiver, allelochemicals are further divided into allomones, kairomones, synomones, and apneumones. Allomones benefit only the emitter. For example, the entomopathogenic fungus *Entomophthora muscae* releases sesquiterpenes that attract healthy *Musca domestica* males to infected females, promoting spore transmission to new hosts (Naundrup et al. 2022). Kairomones benefit only the receiver. Haematophagous flies, such as stable flies, are attracted to animal-derived odours that guide them to blood sources (Getahun et al. 2020). Synomones benefit both emitter and receiver. House flies, lesser house flies (Cook et al. 2020; Dag and Gazit 2000), and stable flies (Tawich et al. 2021) feed on floral nectar while aiding in pollen transfer. Apneumones are chemical cues released by nonliving matter that provide useful information to the receiving organism. Most odours from decomposing material arise from bacterial and fungal activity that converts organic tissues into volatile compounds such as putrescine, cadaverine, hydrogen sulfide, dimethyl disulfide, and dimethyl trisulfide. Although microbes generate these volatiles, the decaying substrate itself is nonliving and can be viewed as the apneumone source from the receiver’s perspective. For example, rotting plant or animal material emits volatile fatty acids, ammonia, and sulfides that attract blowflies (Johansen et al. 2014). Similarly, ethanol and acetic acid from fermenting fruits attract *Muscina stabulans* and *Fannia canicularis* (Landolt et al. 2015), where the nonliving substrate acts as the cue source. However, when microbes benefit from attracting insects, such as through spore dispersal, these odours may instead function as kairomones or synomones. Thus, whether decomposition odours are classified as apneumones depends on whether the decaying substrate or its microbial community is regarded as the emitter.

### Odour Detection in Insects

#### Morphology of the Olfactory System

**Fig. 3:**
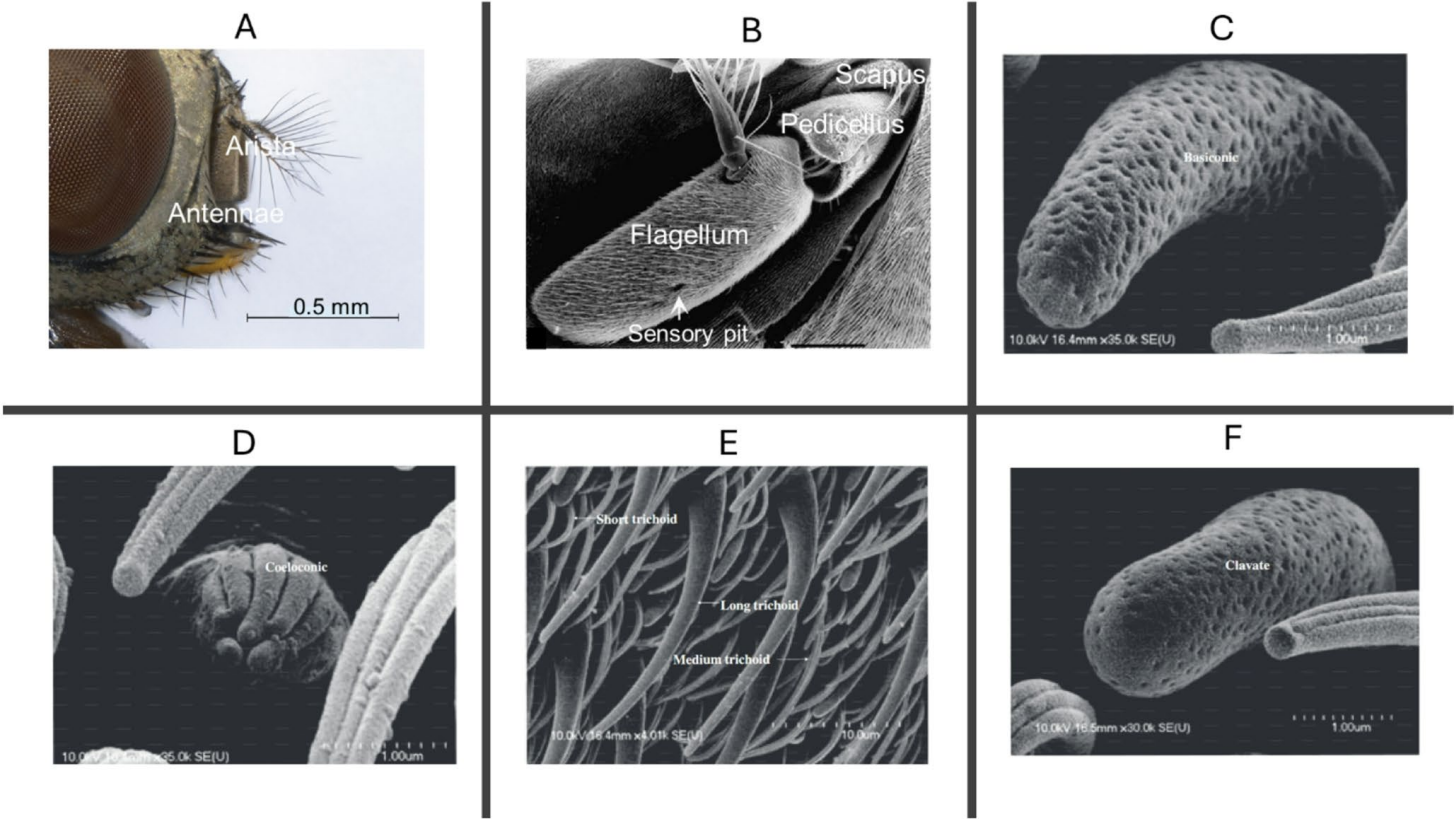
Insect olfactory organs and sensilla types. **A** and **B** Antennal structure of dipteran flies showing the scapus, pedicellus with Johnston’s organ, and funiculus bearing sensilla and the arista. Major sensilla types identified in stable flies, including **C** basiconic, **D** coeloconic, **E** trichoid, and **F** clavate sensilla, which house olfactory sensory neurons detecting volatile semiochemicals. Photo sources: Desquesnes et al. (2019); Tangtrakulwanich et al. (2011)

To detect semiochemicals, insects rely on sensory systems located on the antennae, mouthparts, wings, legs, and ovipositors (Ali and Galizia 2015; Hansson and Stensmyr 2011). Among these organs, the antennae serve as the main structures for detecting volatile cues (Olsson and Hansson 2013). In Diptera, the antenna (Fig. 3A and B) consists of three segments: the basal scapus attached to the head, the pedicellus housing Johnston’s organ, and the funiculus, which bears numerous hairlike sensilla (Guidobaldi et al. 2014; Hansson and Stensmyr 2011; Huotari 2004). The funiculus, which is primarily olfactory and contains sensilla housing olfactory sensory neurons (OSNs) expressing odorant receptors to detect airborne volatiles (Onagbola and Fadamiro 2008), is also the segment from which the arista arises.

Based on external morphology such as length and pore density, sensilla are classified into several types. In *Musca domestica*, *Chrysomya megacephala*, and *Lucilia cuprina*, five types, including trichoid, basiconic, coeloconic, styloconic, and sensory pit, have been described using scanning electron microscopy (Hassan et al. 2013; Li et al. 2016; Smallegange et al. 2008; Sukontason et al. 2004). In *Stomoxys calcitrans*, four types, basiconic (Fig. 3C), coeloconic (Fig. 3D), trichoid (Fig. 3E) and clavate (Fig. 3F), have been reported (Tangtrakulwanich et al. 2011), which are also found in lesser house flies (Zhang et al. 2013). In *Haematobia irritans*, White and Bay (1980) identified grooved sensilla and three morphological types of thin-walled multiporous sensilla, along with thick-walled ones. However, in *Musca sorbens*, *M. autumnalis*, *M. crassirostris*,* Muscina stabulans* and *M. levida*, the number and ultrastructure of antennal sensilla remain undescribed, offering new avenues for comparative and functional studies.

Functionally, sensilla are classified by the odorants detected by their OSNs. Basiconic sensilla typically respond to food-related volatiles (Hallem and Carlson 2006; Keesey et al. 2015; Mansourian et al. 2018). Trichoid sensilla detect pheromones (Khallaf et al. 2021; Kurtovic et al. 2007), while coeloconic sensilla respond to acids, amines, and water vapour (Prieto-Godino et al. 2017; Vulpe et al. 2021; Vulpe and Menuz 2021; Yao et al. 2005). Styloconic sensilla are associated with hygro and thermoreception (Dong et al. 2021), though the role of clavate sensilla remains unclear. A single sensillum type may include several subtypes that differ in size and odorant specificity. For example, Getahun et al. (2020) stimulated basiconic sensilla of *S. calcitrans* with 31 camel-derived odorants and identified 21 distinct subtypes, each with unique response profiles. This study provided the first evidence of odour coding in a filth fly species and underscored the need to extend similar analyses to other filth fly species.

#### Physiology of the Insect Olfactory System

**Fig. 4:**
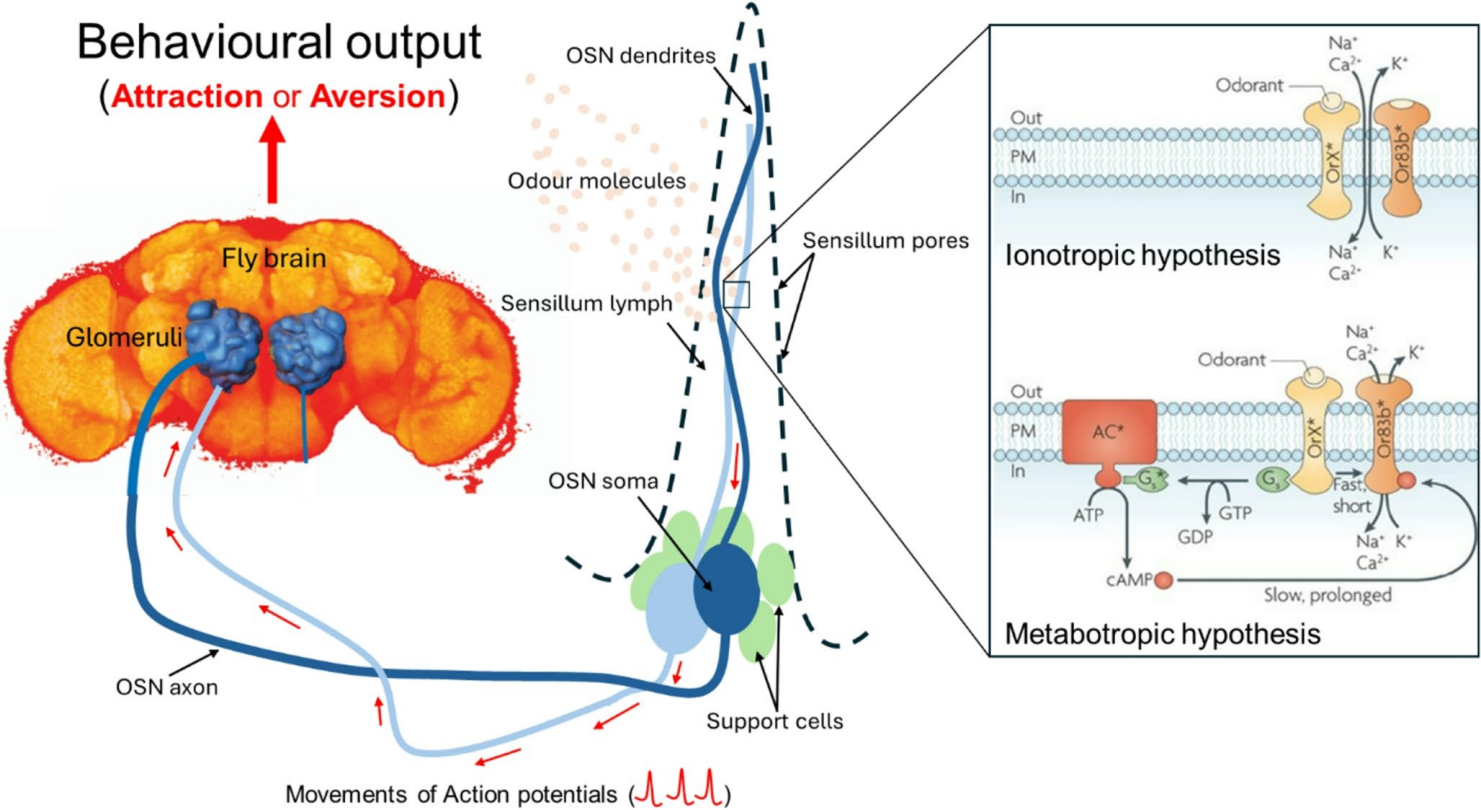
Schematic representation of the physiological basis of odour perception in insects. Odour molecules enter through sensillum pores, bind to odorant-binding proteins (OBPs), and activate olfactory receptors (OR–Orco complex) on sensory neurons. Signal transduction via ionotropic or metabotropic pathways generates action potentials that reach the antennal lobe, where information is processed and sent to higher brain centres controlling learned and innate behaviours linked to odour-guided responses (attraction or aversion). The insect brain image was adapted from Jefferis (2005) while the image illustrating the mechanotropic and the ionotropic mechanisms of odour transduction is adapted from Kaupp (2010)

The perception of an ecologically relevant odour starts when the odour molecule is intercepted by an olfactory sensillum (Fig. 4). It then passes through a sensillum pore and reaches the lymph, where it is transported by an odorant binding protein (OBP) to the dendritic membrane of an olfactory sensory neuron (OSN), where stimulus transduction may occur (Tsuchihara et al. 2005). Signal transduction is initiated by the odour binding to its receptor, which then triggers a cascade of events inside the dendrite, leading to nerve cell activity that results in an electrical signal referred to as an action potential (Leal 2004; Pelosi et al. 2006). There is still a controversial discussion of how odour signals are transduced into the electrical signal. Some authors support the direct odour-gated fast ionotropic hypothesis, suggesting that once an odour molecule binds to the complex odorant receptor (OR)-odorant coreceptor (Orco), the odour molecule initiates a conformational change in the OR that opens a channel in its structure. This leads to an influx of cations (Ca^2+^, K^+^ and Na^+^) into the neuron that generates a fast ionotropic current due to rapid depolarisation of the neuron membrane (Nakagawa et al. 2005; Sato et al. 2008). Others support the metabotropic hypothesis postulating that the liaison between the odour and the OR-Orco triggers a cascade of reactions that involve the activation of the guanine nucleotide binding protein (G-protein) and adenylyl cyclase enzyme that leads to the production of the second messenger cyclic adenosine monophosphate (cAMP), which indirectly leads to depolarisation of the neuron membrane by activating ion channels separate from the OR (Getahun et al. 2016; Stengl and Funk 2013; Wicher et al. 2008). The action potential from the transduction goes through the OSN axon and reaches the primary olfactory centre, the antennal lobe (AL), formed by spherical structures called glomeruli (Meyer et al. 2013). This structure is usually innervated by axons from OSNs that express the same type of ORs (Couto et al. 2005; Vosshall et al. 2000). Overall, most Diptera species have 50–70 glomeruli; a number that is species- and sex-specific (Guidobaldi et al. 2014). When it reaches the glomeruli, the action potential is transferred from the OSN axons (via neurotransmitters) to the dendrites of projection neurons (PNs) that transmit the processed signal to the higher brain, namely the mushroom body and the lateral horn (Vosshall and Stocker 2007) that respectively play a substantial role in maintaining insect learning (Heisenberg 2003) and innate (Das Chakraborty et al. 2022) behaviours. In the higher brain, the action potentials are translated into neuronal representations of various stimulus parameters, such as, e.g., odour identity, odour concentration, and/or hedonic valence (pleasant vs. unpleasant) (Haddad et al. 2008; Knaden et al. 2012). This constitutes the basis of behavioural output such as the acceptance of an oviposition site, food source, sexual partner, or the avoidance of a pathogen, predator or parasitoid (Kreher et al. 2008). In insect pest management, the identification of the odourants mediating these behavioural outputs is crucial, as they can be used as attractants or repellents.

## Exploitation of Odour Cues for the Control of Filth Flies

**Fig. 5:**
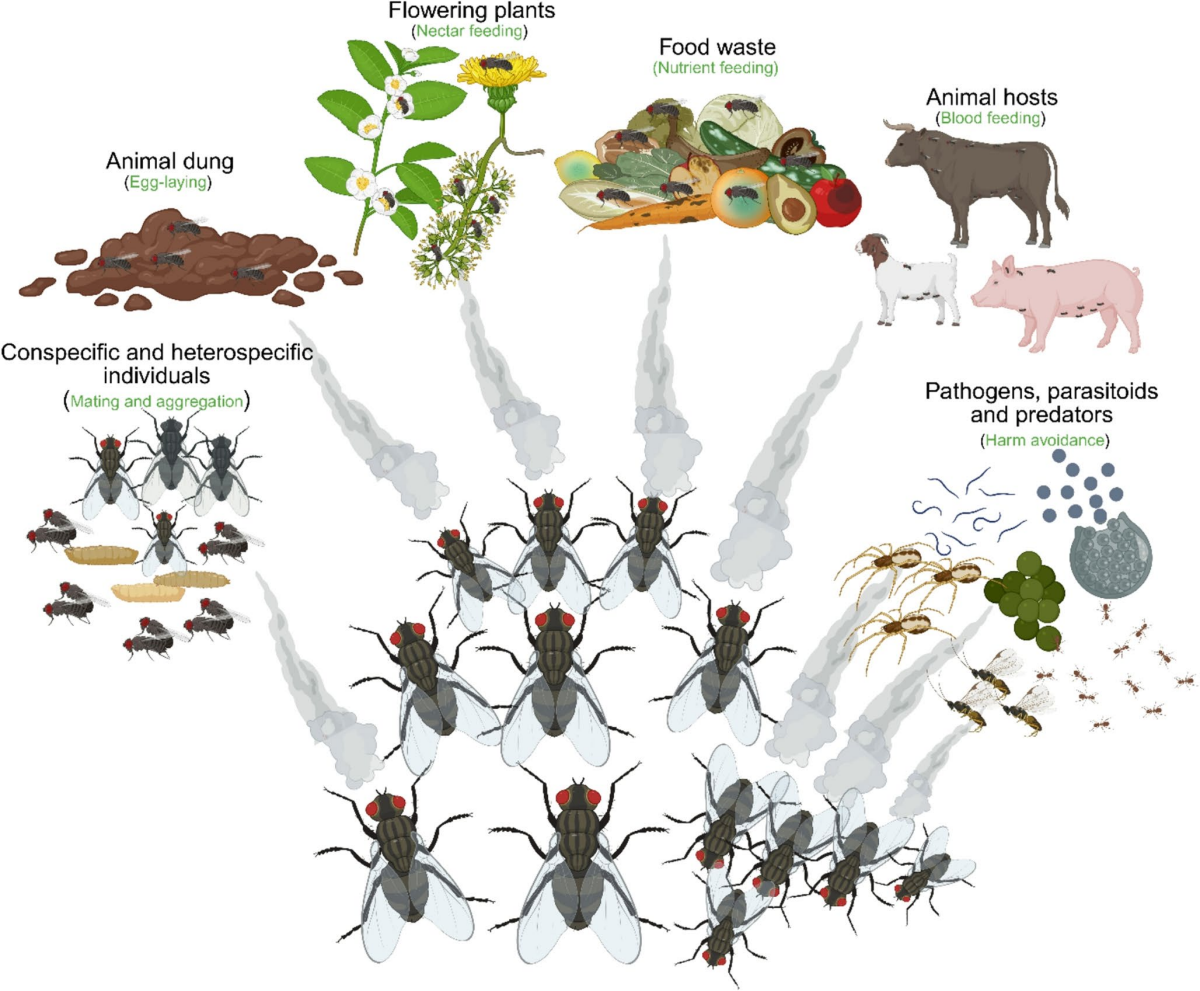
Sources of odour cues used by filth flies to guide key behaviours, such as feeding, mating, egg-laying, and avoiding danger. These cues originate from food sources, oviposition substrates, conspecific and heterospecific individuals, and harmful organisms

Odour cues that filth flies use to perform key behaviours such as feeding, mating, egg laying, and avoiding danger originate from food sources, oviposition substrates, conspecific and heterospecific individuals, and harmful organisms (Fig. 5). When successfully isolated, identified, and formulated, these cues can be used as control tools.

### Odour From Food Sources

Volatile organic compounds (VOCs) that filth flies use to locate food sources are mainly kairomones and synomones, respectively, emitted by animal and plant hosts.

Obligate haematophagous filth flies primarily depend on animal hosts for blood meals that facilitate sexual maturation and reproduction. To find these hosts, they utilise kairomones released from breath, skin, urine, and faeces. Schofield et al. (1995) demonstrated that 1-octen-3-ol and 3-methylphenol produced by animal hosts triggered strong electroantennogram (EAG) responses in *Stomoxys calcitrans*. Nayani et al. (2023) found that volatiles from Staphylococcus microbes on bovine skin attracted *S. calcitrans* to the host. Using cows, Jeanbourquin and Guerin (2007) identified dimethyl trisulfide, butanoic acid, and p-cresol from rumen emissions as stimulants of *S. calcitrans* antennae and organisers of upwind flight in wind tunnel tests. Similarly, Getahun et al. (2020) recorded single sensillum responses to camphor, naphthalene, p-cresol, α-pinene, and 1-octen-3-ol derived from camel breath, skin, urine, and faeces, which boosted field trap catches of *S. calcitrans* when employed in monoconical traps. Since *S. calcitrans* feeds on various animals, including rats, rabbits, goats, and birds (Lehane 2005; Pitzer et al. 2011), identifying odours from these hosts remains essential for creating species-specific attractants. *Haematobia irritans* and *Musca crassirostris* mainly feed on cattle but may also attack camels, deer, dogs, horses, goats, sheep, and even tigers (Brewer et al. 2021; Desquesnes et al. 2019). In *H. irritans*, Birkett et al. (2004) showed that 1-octen-3-ol and 6-methyl-5-hepten-2-one from heifer skin and urine activated antennal responses and increased upwind flight, whilst also reducing fly populations in field conditions. For *M. crassirostris*, animal-derived attractants have not yet been identified.

Besides blood meals, obligate haematophagous filth flies also require sugar from fruits and flowers for energy. In Mali, *Stomoxys* flies were observed feeding on floral and fruit sources (Müller et al. 2012), likely guided by plant volatiles. For example, γ-terpinene from *Schinus terebinthifolia* strongly attracted stable flies and enhanced trap catches when incorporated into monoconical traps (Tawich et al. 2021). Similar investigations are needed for *H. irritans* and *M. crassirostris* flies.

Facultative filth flies exhibit a more diverse range of feeding habits. They feed on organic waste, excreta, carcasses, wound secretions, milk, and nectar. These feeding behaviours are governed by volatiles emitted from these substrates. Dar et al. (2020) showed that bottle traps baited with fermented sugar and milk solutions captured more *Musca domestica*, *M. sorbens*, and *M. automnalis*. Mulla et al. (1977) identified trimethylamine, ammonia, indole, and linoleic acid as attractants for *M. domestica*. Quinn et al. (2007) reported that hexane and diethyl ether extracts of blackstrap molasses released several odorants acting as feeding attractants. Honeydew, the sugary excreta of sap-feeding insects, emits benzaldehyde and (Z)−3-hexenyl acetate, which stimulate *M. domestica* antennae and attract flies under semifield conditions (Hung et al. 2015, 2020). Indole from *Periploca laevigata* flowers also attracts *M. domestica* flies (Zito et al. 2015). Guarino et al. (2022) showed that the window fly blade traps loaded with nine odorants derived from sweet and fetid food sources (terpinolene, α-terpinene, linalool, acetic acid, butyric acid, isovaleric acid, hexanoic acid, indole, and dimethyl trisulfide) significantly increased *M. domestica* captures. Likewise, traps baited with acetic acid and ethanol, two sugar fermentation volatiles, enhanced catches of *Fannia canicularis* and *Muscina stabulans* flies (Landolt et al. 2015).

In *Lucilia cuprina*, electrophysiological work has revealed that female antennae contain olfactory receptor neurons (ORNs) tuned to host-related volatiles, guiding the development of attractant baits. Park and Cork (1999) identified three types of neurons: “Oct-best” (responsive to 1-octen-3-ol), “DMDS-best” (dimethyl disulfide), and “PE-best” (2-phenylethanol). Oct-best neurons showed dose-dependent firing, while carboxylic acids and 3-methylindole inhibited spontaneous activity, indicating fine-tuned neural sensitivity to host and decomposition volatiles. These findings informed later bait development. Urech et al. (2004) showed that synthetic blends containing sulfur volatiles (2-mercaptoethanol, indole, butanoic or pentanoic acids, sodium sulfide) attracted 5–20 times more *L. cuprina* than liver-based baits, proving the value of reformulated attractant blends. Yan et al. (2018) further demonstrated that dimethyl trisulfide (DMTS) elicited strong antennal and behavioural responses, particularly in gravid females, identifying it as a promising trap lure. Recent molecular data reinforce these behavioural observations. Wulff et al. (2024) identified female-biased odorant receptors (e.g., LcupOR46) and an ammonia transporter (AMT_Rh50) expressed in *L. cuprina* antennae, suggesting roles in host detection and offering molecular targets for designing more selective semiochemical lures.

Several studies have identified food-derived odours that are useful for controlling *Chrysomya megacephala*. Using decomposing animal materials as models, researchers found that volatile organic compounds (VOCs) such as phenol, 2-nonanone, dimethyl sulfide, and indole emitted from decomposed shrimp were strongly attractive to *C. megacephala* (Silva et al. 2019). Similarly, in wind-tunnel assays, pork viscera decomposed for one day elicited over 90% attraction, outperforming beef liver, a commonly used bait (Bunchu et al. 2008). Field studies also confirmed that traps baited with tainted beef offal captured large numbers of flies across diverse habitats, indicating the effectiveness of such host-derived volatiles under natural conditions (Sontigun et al. 2018). Collectively, these findings highlight key host-derived compounds—especially phenol, indole, dimethyl sulfide, and 2-nonanone—as promising candidates for developing odour-based lures and attract-and-kill traps targeting *C. megacephala*. Studies on *Calliphora vicina* have identified several food-derived odours that elicit strong behavioural and physiological responses, making them useful for control applications. Aak et al. (2010) reported that *C. vicina* was highly attracted to volatiles from decomposing animal material such as liver, fish, and dead mice, compared to clean air or individual synthetic compounds. Among tested volatiles, dimethyl trisulfide (DMTS) was the most attractive single compound, while a blend of DMTS, mercaptoethanol, and o-cresol significantly enhanced fly captures in both wind-tunnel and field experiments. The same authors found that the response varied with the fly’s physiological state, with gravid females showing stronger attraction to these cues. These odourants originate from microbial decomposition of animal tissues and are therefore considered host-associated kairomones that guide *C. vicina* to suitable feeding and oviposition substrates.

Overall, research has advanced the understanding of semiochemical cues mediating food selection in filth flies, yet several questions remain unresolved, and their investigation is warranted. (1) **Host-specific odour signatures**: What volatile profiles distinguish preferred animal hosts among obligate haematophagous filth flies (*Stomoxys*, *Haematobia*, and *Musca crassirostris*)? How do microbial communities associated with different host species influence these odour signatures, and can host-specific kairomones be identified to develop selective attractants? (2) **Role of microbial symbionts in odour production**: Which bacterial taxa are primarily responsible for producing key kairomones such as p-cresol, indole, or DMTS? To what extent does the composition of skin or gut microbiota affect volatile emission and fly perception, and could microbial manipulation (e.g., through probiotics or inhibitors) suppress the cues that attract flies? (3) **Sex- and stage-specific olfactory tuning**: Do males and females detect or prioritise distinct sets of host-derived volatiles, and how does reproductive or nutritional status (e.g., gravid versus non-gravid) modify antennal sensitivity? What molecular mechanisms, such as receptor gene expression changes, underlie these differences? (4) **Molecular basis of odour detection**: Which specific odorant receptors and transporters mediate responses to host volatiles in *Stomoxys*, *Haematobia*, *Musca*, *Lucilia*,* Muscina*,* Faniia*,* Calliphora* and *Chrysomya* species, and how conserved are these receptors across filth fly lineages? Can receptor–ligand mapping inform the rational design of next-generation synthetic lures? (5) **Integration of plant and animal cues**: How do filth flies integrate signals from both plant and animal hosts when foraging, and do plant-derived volatiles enhance or inhibit attraction to animal kairomones? Could combined plant–animal odour blends yield more effective baits? (6) **Chemical ecology of neglected species**: Why do attractant cues for *Musca crassirostris* remain poorly characterised despite its veterinary importance, and are its sensory preferences distinct from those of other haematophagous or facultative filth flies? (7) **Environmental modulation of semiochemical responses**: How do temperature, humidity, and host diet alter the emission and detectability of kairomones, and do seasonal changes in odour profiles affect trap performance under field conditions? (8) **Behavioural ecology in mixed fly communities**: How do multiple filth fly species sharing the same habitat interact at odour sources, and could interspecific odour masking or signal interference influence trap selectivity? (9) **From discovery to application**: What are the optimal release rates and formulations for identified volatiles under tropical conditions, and can synthetic lures effectively replace decomposing substrates without reducing catch efficiency?

Addressing these hypotheses will advance the mechanistic understanding of olfactory-driven behaviours in filth flies and support the development of ecologically sound odour-based control tools.

### Odour Cues From Oviposition Substrate Sources

Odour cues used by gravid female filth flies to select suitable oviposition sites originate from organic wastes, microbes, and developmental stages (eggs, larvae, pupae) of conspecific and allospecific species. Depending on the substrate’s suitability for offspring development, these cues can function as attractants or repellents.

#### Cues From Organic Wastes

Organic wastes such as animal dung, carrion, and fermented plant matter emit odorants that guide gravid females during oviposition. For example, females of *Musca domestica* readily oviposit on fermented pig manure, from which Cossé and Baker (1996) identified nine electrophysiologically active compounds using gas chromatography–electroantennography (GC–EAD). In wind-tunnel assays, mixtures of three (3-methylindole, butanoic acid, and 3-methylbutanoic acid) or seven (3-methylindole, butanoic acid, 3-methylbutanoic acid, dimethyl trisulfide, indole, benzene ethanol, and phenol) volatiles elicited strong upwind flight responses. Tang et al. (2016) further showed that ethyl palmitate, ethyl linoleate, methyl linoleate, and linoleic acid emitted from fermented wheat bran activated the antennae of *M. domestica* and acted as oviposition attractants, increasing egg deposition when added to non-preferred substrates. Females of this fly also oviposit on chicken manure, maize bran, and soybean bran (Ganda et al. 2019), as well as on fresh horse (Machtinger et al. 2014) and cattle dung (Broce and Haas 1999). However, the odorants mediating these behaviours remain poorly characterised, as in *Haematobia irritans* (Kuramochi 2000) and *Musca crassirostris* flies (Desquesnes et al. 2019). Both of these species lay eggs primarily on fresh cattle dung. Similarly, the chemical cues guiding oviposition in *Musca autumnalis* and *Fannia canicularis*, which prefer fresh cattle dung (Dougherty and Knapp 1994; Teskey 1969) and fermented poultry manure (Murillo et al. 2021), respectively, are still unknown.

In contrast, *Stomoxys calcitrans* prefers aged dung for oviposition (Albuquerque and Zurek 2014; Broce and Haas 1999; Romero et al. 2006). Tangtrakulwanich et al. (2015) identified methoxy-phenyl oxime, phenol, *m*-cresol, *p*-cresol, and 4-ethylphenol as attractants from cattle manure slurry, with binary blends significantly increasing trap catches in the field. Baleba et al. (2019) further demonstrated that *S. calcitrans* oviposits preferentially on donkey and sheep dung, which enhances offspring fitness. Through dynamic headspace collection and GC–MS combined with a random forest algorithm (Breiman 2001), β-citronellene and carvone were identified as key volatiles from donkey and sheep dung, respectively. Both compounds stimulated oviposition and increased trap catches, highlighting the potential of semiochemical-based control. Other substrates, including decomposing sugarcane stems (Cançado et al. 2013) and vinasse, a sugarcane–ethanol by-product, also attracts *S. calcitrans* (Jelvez Serra et al. 2017). GC–EAD analysis revealed eleven antenna-activating volatiles, such as butanoic acid, phenethyl alcohol, and cinnamic aldehyde, though their behavioural effects remain untested. *S. calcitrans* also uses vegetable residues such as celery, brassicas, lettuce, and eggplant for oviposition (Cook et al. 2018), suggesting diverse chemical cues across substrates.

*Musca sorbens* preferentially lays eggs on human faeces (Emerson et al. 2001). GC–EAD analyses identified several antenna-stimulating compounds, including 3-ethylpentane, 2-methylpropanoic acid, butanoic acid, cresol, indole, and dimethyl tetrasulfide (Robinson et al. 2020), though their behavioural functions as oviposition cues in the laboratory and field conditions remain undetermined.

Studies on *Lucilia sericata* provide clear evidence that organic wastes influence oviposition decisions. Brodie et al. (2014) showed that gravid females responded strongly to incised rat carrion through a bimodal cue complex of visual and olfactory signals. GC–EAD analyses identified nine key volatiles—phenol, *p*-/*m*-cresol, guaiacol, dimethyl trisulfide (DMTS), phenylacetaldehyde, (E)−2-octenal, nonanal, and tetramethyl pyrazine—eliciting antennal responses, with DMTS alone sufficient to attract gravid females. Subsequent work revealed that physiological state modulates semiochemical preference: protein-fed gravid females preferred carrion, while protein-starved females responded equally to faeces and carrion (Brodie et al. 2016). Faecal headspace analysis identified odorants such as indole, phenol, *m/p*-cresol, and 1-octen-3-ol that, when blended, reproduced faecal attraction. These findings demonstrate that specific volatiles, particularly DMTS and cresols, mediate oviposition in *L. sericata*. However, whether other organic wastes such as dung, compost, or fermented plants emit comparably attractive cues remains untested. The relative importance of stimulants versus attractants, and the influence of microbial composition, humidity, decay stage, and temperature on odour emission, are also unresolved.

In contrast, there is limited chemical evidence that animal dung or fermented plant materials emit oviposition stimulants for *Chrysomya megacephala*. Esser (1991) reported strong attraction of gravid females to fish during the salting stage of processing, but did not identify the odorants involved, suggesting that volatiles from decomposing fish may serve as oviposition cues. It remains unclear whether volatiles from dung or fermented plant matter induce oviposition in *C. megacephala* or function merely as general attractants.

#### Cues From Microbes, Parasitoids and Predators

The microbial community in a potential oviposition substrate strongly influences egg-laying decisions in filth flies. Gravid females detect and select suitable sites using microbially derived odourants that signal conditions favourable for offspring development. For example, *Proteus mirabilis* produces volatiles such as indole and isobutylamine that attract and stimulate oviposition in *Lucilia sericata* (Ma et al. 2012; Tomberlin et al. 2012, 2017). Similarly, *Citrobacter freundii* induces strong oviposition responses in stable flies, likely through the release of highly attractive odours (Romero et al. 2006). In contrast, the proliferation of the symbiont *Klebsiella oxytoca* on house fly eggs suppresses further egg-laying in this species (Lam et al. 2007). Future studies analysing the headspace of *C. freundii* and *K. oxytoca* cultures could help identify the specific volatiles driving these behavioural effects. Current evidence shows that *C. freundii* filtrates contain mainly ammonia (Robacker and Bartelt 1997), a known attractant for many insects such as *Aedes aegypti* (Geier 1999) and fruit flies *Anastrepha ludens* and *A. suspense* (Thomas et al. 2008). In *Bactrocera dorsalis*, bacterial isolates such as *Providencia* sp. and *Klebsiella* sp. from egg surfaces produce β-caryophyllene, which repels gravid females from infested fruits (Li et al. 2020). In *Chrysomya megacephala*, research has mainly focused on bacterial diversity across developmental stages and within pupae (Wang et al. 2018; Xu and Wang 2022). However, no study has yet linked microbial volatiles to oviposition behaviour in this species. Whether volatiles emitted by bacterial communities in oviposition substrates influence egg-laying decisions in *C. megacephala* and other filth flies, such as *Calliphora vicina*, *Musca autumnalis*, *M. sorbens*, *M. stabulans*, *M. levida*, *F. canicularis*, *H. irritans*, and *M. crassirostris*, remains to be tested.

In addition to bacteria, entomopathogenic fungi in oviposition substrates can also influence the egg-laying behaviour of filth flies. Machtinger et al. (2016) found that *Metarhizium brunneum* (Metsch.) (Ascomycota: Hypocreales), when applied to oviposition substrates, significantly deterred oviposition by house flies and stable flies. Similarly, Baleba et al. (2021) reported that gravid females of *Stomoxys calcitrans* laid fewer eggs on rabbit dung treated with *Metarhizium anisopliae* than on untreated dung. Since both *M. brunneum* (Hummadi et al. 2021) and *anisopliae* (Bojke et al. 2018) emit diverse odorants, identifying those responsible for oviposition avoidance would be valuable. Extending such investigations to other filth fly species could reveal novel oviposition repellents. For instance, Lam et al. (2010) showed that odorants produced by harmful fungi (*Phoma* spp., *Fusarium* spp., and *Rhizopus* spp.) growing on chicken dung mediated oviposition avoidance in gravid females of *Musca domestica*. Using coupled gas chromatography–electroantennographic detection (GC–EAD), the authors identified five compounds—dimethyl trisulfide, 2-phenylethanol, citronellal, norphytone and an unknown compound—that elicited antennal responses. In behavioural assays, dimethyl trisulfide and 2-phenylethanol significantly reduced oviposition by *M. domestica*. Although the persistence of these effects under natural conditions was not tested, the study highlights how fungal odorants can be exploited to identify repellents. Beyond these examples involving *M. domestica* and *S. calcitrans*, no studies have yet isolated specific fungal volatiles that attract or deter oviposition in other filth fly species.

The presence of entomopathogenic nematodes (EPNs) also offers great opportunities to discover oviposition-repellent (s) for filth flies. When they are present in substrates, they can deter egg-laying in flies. EPNs are symbiotically associated with bacteria that produce odourants responsible for this deterrence. For instance, Gulcu et al. (2012) found that *Chrysomya albiceps* (Diptera: Calliphoridae) gravid females avoid depositing eggs on meat treated with *Photorhabdus luminescens*, a symbiotic bacterium of the EPNs species belonging to the genus *Heterorhabditis*. The authors claimed that odours produced by *P. luminescens* mediate this avoidance. In *Lobesia botrana* (Lepidoptera: Tortricidae), the odours emitted by *Xenorhabdus nematophila* and *Photorhabdus laumondii*, bacterial symbionts of the nematodes in the families Steinernematidae and Heterorhabditidae, respectively, repel gravid females and deter the feeding activity of larvae (Vicente-Díez et al. 2023). Although these authors did not identify the specific odorants responsible for the behavioural deterrents, their work supports the hypothesis that olfactory cues emitted by bacterial–nematode symbionts may signal pathogen presence and discourage oviposition by gravid filth flies on contaminated substrates. Testing this hypothesis in filth flies using the classical chemical ecology approaches, i.e. analytical chemistry (headspace volatile collection, gas chromatography coupled with mass spectrophotometry), electrophysiological (electroantennography, single sensillum recording), and oviposition and field trapping assays, could lead to the discovery of oviposition repellent(s). For illustration, Kong et al. (2022) used this approach to identify 2-hexynoic acid, an odour produced by symbiotic bacteria of *Steinernema* and *Heterorhabditis* nematode species, as the molecule which prevents *Spodotera frugiperda* (Lepidoptera: Noctuidae) larvae from locating and feeding on corn leaves.

#### Cues From Conspecific and Heterospecific Individuals

The presence of eggs, larvae, or pupae on food or breeding sites can strongly affect the feeding and oviposition behaviour of both conspecific and heterospecific adult flies. The magnitude of this effect often depends on the density of these preimaginal stages and the odours they emit (Bentley and Day 1989). For example, Bradley and Sheppard (1984) found that house flies avoided ovipositing in poultry manure heavily infested with black soldier fly (*Hermetia illucens*) larvae, suggesting interspecific chemical communication via allomones. Likewise, Baleba et al. (2020) reported that gravid females of *Stomoxys calcitrans* avoided substrates containing high densities of conspecific and house fly larvae, indicating larval odours may act as repellents. Blowflies exhibit more complex patterns: *Chrysomya megacephala* females prefer substrates containing small numbers of conspecific eggs, which signal freshness, but avoid large egg masses that indicate intense competition (Lima and Von Zuben 2016). When larvae of competing species, such as *C. rufifacies*, are present, *C. megacephala* females avoid these substrates, exhibiting species-specific risk avoidance (Yang and Shiao 2012). However, knowledge on how chemical cues from eggs, larvae, or pupae shape oviposition decisions in filth flies remains limited. Research on other dipterans shows potential for discovering such compounds. For instance, larvae of *Aedes aegypti* produce n-heneicosane, which deters oviposition at concentrations of 100–1000 mg/L, while the same compound repels *Aedes albopictus* at concentrations of 30–200 ppm (Mendki et al. 2000; Seenivasagan et al. 2009). Identifying comparable odourants in filth flies could open new avenues for developing repellents and improving their integrated management strategies.

Additionally, the presence of predators and parasitoids in a breeding substrate can trigger avoidance behaviour in flies through the volatiles they emit. Several studies show that flies detect and avoid odour cues from their natural enemies. For example, *Drosophila melanogaster* larvae and ovipositing females actively avoid areas containing semiochemicals released by parasitoid wasps such as *Leptopilina boulardi* and *L. heterotoma*, including the wasp sex pheromone iridomyrmecin, which signals high parasitism risk (Ebrahim et al. 2015). Similarly, studies on *Bactrocera tryoni* demonstrate that this species detects olfactory cues from predators and adjusts its behaviour to reduce predation risk. Flies exposed to predator odours, such as those from spiders and ants, show reduced foraging, mating, and oviposition, indicating that chemical cues alone can trigger defensive behavioural changes (Kempraj et al. 2020; Rathnayake et al. 2019). These findings suggest that volatiles emitted by parasitoids and predators mediate risk-avoidance behaviour in flies. In contrast, direct evidence that filth flies use predator- or parasitoid-emitted volatiles to avoid harm remains scarce. Targeted experiments isolating these volatiles and testing avoidance responses in these flies are therefore needed. In a management context, predator- and parasitoid-derived volatiles could serve as natural repellents or deterrents. If flies recognise and avoid odours associated with predation or parasitism, synthetic analogues of these compounds could be developed to drive flies away from livestock facilities, waste sites, and human habitats. Such tools could provide environmentally friendly alternatives to insecticides and improve integrated pest management when combined with attract-and-kill or push–pull systems.

### Pheromones

Pheromones play a major role in pest management strategies such as mating disruption, mass trapping, attract-and-kill, and push–pull systems. In filth flies, research has mainly focused on sex pheromones. A breakthrough in *Musca domestica* management was the discovery of (Z)−9-tricosene, a sex pheromone isolated from the cuticle and faeces (Carlson et al. 1971). Male flies become sexually stimulated when exposed to this compound. When used in traps, (Z)−9-tricosene significantly increases capture rates. Carlson and Beroza (1973) showed that sticky panels, flypaper strips, and electric grid traps baited with (Z)−9-tricosene caught more flies than unbaited controls. Similarly, on a swine farm in Florida, Morgan et al. (1974) reported that sugar bait containing trichlorfon and 125 mg of (Z)−9-tricosene captured more male and female flies than baits containing 25, 5, or 1 mg of the pheromone. Kannan et al. (2020) found that Barrix Domo traps lured with 25–30 g of (Z)−9-tricosene gel caught significantly higher numbers of *M. domestica* flies in India. In *Fannia canicularis*, (Z)−9-pentacosene elicits copulatory responses in males. Field traps baited with this pheromone increased male catches (Uebel et al. 1978a, b). In *Musca autumnalis*, extracts from virgin females attract males in Y-tube olfactometer assays (Chaudhury et al. 1972). Uebel et al. (1975a, b) identified (Z)−14-nonacosene, (Z)−13-nonacosene, and (Z)−13-heptacosene as compounds that trigger male mating responses, with (Z)−14-nonacosene being the most active (Sonnet et al. 1975). In *Stomoxys calcitrans*, a blend of (Z)−9-hentriacontene, (Z)−9-tritriacontene, 13-methyl-1-hentriacontene, and 13-methyl-1-tritriacontene from female cuticular extracts induced copulatory behaviour in males (Sonnet et al. 1979; Uebel et al. 1975a, b). Similarly, in *Haematobia irritans*, a mixture of (Z)−5-tricosene, (Z)−9-pentacosene, and (Z)−9-heptacosene from females stimulated male courtship (Bolton et al. 1980). However, the field efficacy of sex pheromones in *M. automnalis*, *S. calcitrans*, and *H. irritans* remains largely untested. Furthermore, the identification and functional testing of sex pheromones in *Musca. sorbens*, *M. autumnalis*, *Chrysomya megacephala*, *Lucilia. sericata*, *L. cuprina*, and *Calliphora vicina* are still unresolved. Integrating behavioural observations, chemical analyses, electrophysiological assays, and field bioassays will be crucial to elucidate pheromone-mediated mate communication in these species.

In addition to sex pheromones, aggregation pheromones mediate group-forming behaviour observed in several fly species, including the house fly *Musca domestica* (Acree et al. 1959; Barnhart and Chadwick 1953), *Phormia regina* (Dethier 1955), *Lucilia sericata* (Brodie et al. 2015), and *Musca autumnalis* (Teskey 1969). Barnhart and Chadwick (1953) first reported that food previously visited by flies attracted more foraging individuals than identical but non-visited food, describing this collective response as a “fly factor”. The cues responsible for this response may be chemical, visual, acoustic, or tactile, and often originate from the flies themselves (Eichorn et al. 2017) or from their faeces, regurgitate, and deposited eggs (Holl and Gries 2018). Foraging flies are also attracted to aggregation pheromones produced by conspecifics or to odours emitted by bacterial symbionts associated with the flies (Uriel et al. 2020; Venu et al. 2014). Despite extensive research, major gaps persist in understanding and applying the “fly factor” to control filth flies. (1) The specific semiochemicals driving this phenomenon remain unidentified, limiting the development of attractants or repellents. (2) The contribution of fly-associated microbiota to volatile production is poorly understood, though evidence suggests bacterial metabolites play a key role. (3) Studies have largely focused on a few filth fly species, leaving interspecific and cross-attraction mechanisms unexplored. (4) The relative importance of volatile versus contact cues is unclear, as many experiments fail to isolate these components. (5) Quantitative data on dose–response relationships, the number of visiting flies required to elicit attraction, and cue longevity are lacking. (6) The effects of substrate type and decomposition stage on cue strength remain understudied. (7) Laboratory findings are rarely validated under field conditions, limiting their practical relevance. (8) The ecological and non-target consequences of exploiting aggregation cues are not well characterised. (9) Finally, limited efforts have been made to integrate the fly factor into applied management tools such as lures, traps, or push–pull systems.

## Factors to Consider When Establishing Control Strategies Using Odour Cues

**Fig. 6:**
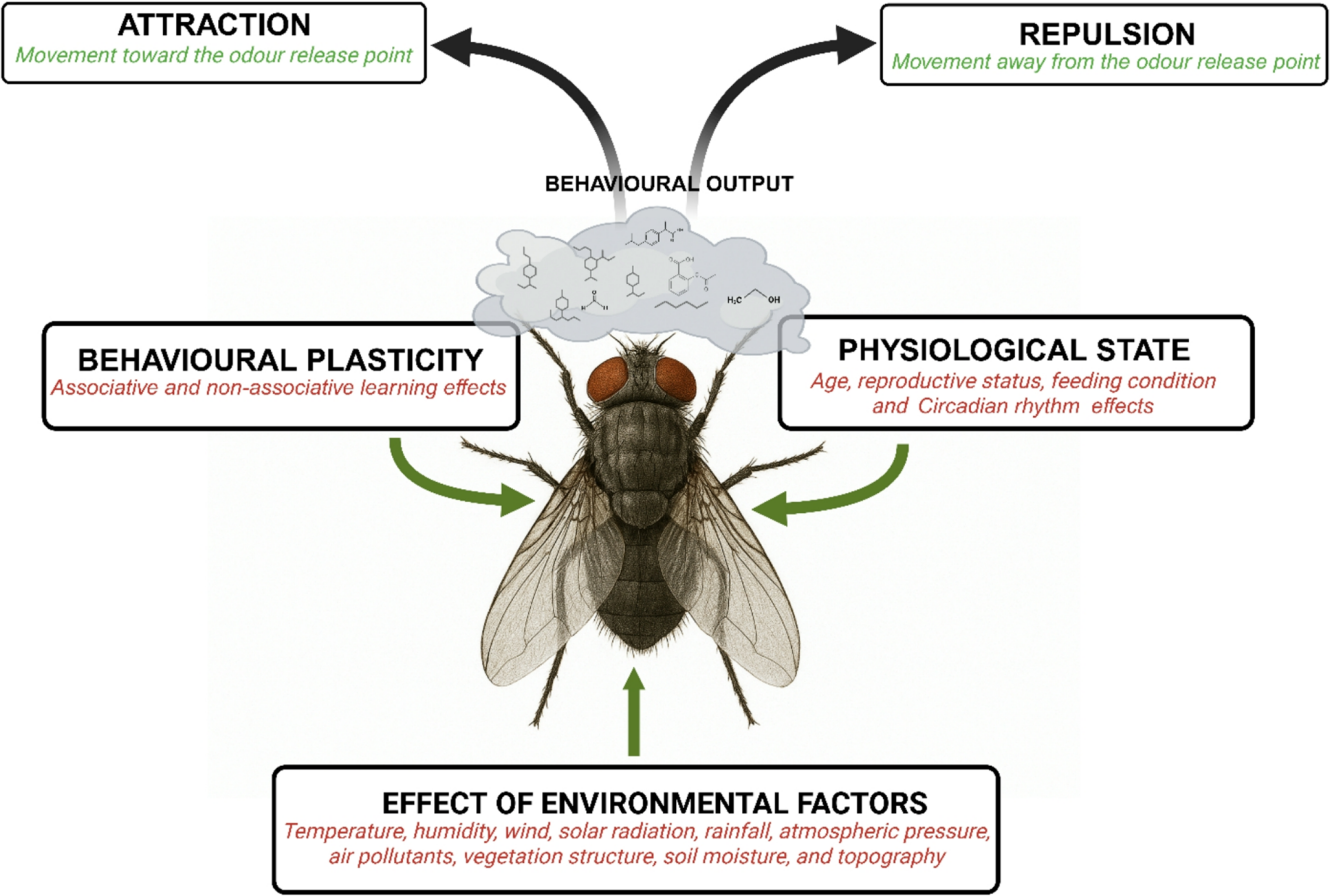
Illustration of the factors influencing effective odour-based monitoring and control strategies, including the insect’s behavioural plasticity, physiological state, and environmental effects on semiochemical emission, dispersion, and perception

To establish effective monitoring and control strategies using odour cues, it is essential to understand the target insect’s behavioural plasticity, physiological state, and how environmental factors influence the chosen semiochemical, its emission, dispersion and perception (Fig. 6).

### Behavioural Plasticity and Habituation in Insect Responses to Odour Cues

Behavioural plasticity, which involves associative and non-associative learning, affects odour-guided behaviour in insects (Reisenman et al. 2016). In natural environments, insects encounter numerous background odours that can affect their responses [reviewed by Schröder and Hilker (2008) and demonstrated by Riffell et al. (2014)]. Here, a form of non-associative learning called behavioural habituation is usually formed owing to the adaptation of the insect olfactory system to the repetitive stimulus from background odours. This leads to the reduction of the behavioural responsiveness of the insect to these odours (Kadohisa and Wilson 2006; Linster et al. 2007). Thus, efficient insect attractants or repellents that are developed in the laboratory may not be effective in field conditions if the targeted insects have already been exposed to them for an extended period. For instance, in *Aedes aegypti*, the previous exposure to DEET (*N*,* N*-Diethyl-*m*-toluamide) leads to the reduction of repellency (Stanczyk et al. 2013). A prolonged exposure to the alarm pheromone in triatomine bugs reduces their repellency behaviour (Minoli et al. 2013). Carbon dioxide (CO_2_) is used by hematophagous insects to find vertebrate hosts (Cozzarolo et al. 2020; Gillies 1980). However, when the natural CO_2_ background is elevated, its ability to attract insects is significantly reduced. Majeed et al. (2014) demonstrated that elevated background levels of CO_2_ hinder the take-off and source contact behaviour of *A. aegypti* by masking the stimulus signal. The authors propose that the vanishing of host-seeking behaviour observed in *A. aegypti* may be attributed to the habituation of neurons in response to the elevated CO_2_ background. Also, it is known that the stimulation of olfactory sensory neurons with high concentrations of odours leads to a complete decline of their action potential amplitude (Ghatpande and Reisert 2011; Reisert and Matthews 1999). As insects can respond to odour mixtures, traps with multi-component odour baits will be more attractive to insects than those baited with a single odourant. For example, triatomines are sensitive to various human odours, including CO_2_, ammonia, lactic and pentanoic acid (Guerenstein and Lazzari 2009). To overcome habituation and sustain insect responsiveness under field conditions, several strategies can be applied: (1) use odour blends targeting multiple sensory pathways by combining host-, food-, and sex-related cues; (2) rotate or refresh semiochemicals periodically to prevent prolonged exposure; (3) integrate olfactory cues with visual, thermal, or auditory stimuli to enhance behavioural responses; (4) optimize release rate and timing to mimic natural emission patterns or align with peak activity periods, using pulsed or intermittent odour release when possible; (5) match odour type with behavioural phase, using sugar cues for newly emerged adults, host cues for reproductively mature individuals, and oviposition cues for gravid females; and (6) reduce background interference by deploying traps in areas with minimal competing odours or upwind from strong odour sources.

### Influence of Physiological State On Insect Responses to Odour Cues

The physiological state of an insect influences how it perceives and responds to semiochemical cues. Factors such as age, reproductive status, feeding condition, and circadian rhythm can modify odour sensitivity and behavioural response.

#### Age

Odour perception in insects often changes with age. Newly emerged female mosquitoes respond weakly to host odours because they first need to locate sugar sources for energy and maturation (Hill and Ignell 2021). At this stage, they are more responsive to plant-derived odours and remain largely unresponsive to human cues until they have fed, mated, and reached reproductive maturity (Foster and Takken 2004; Kemibala et al. 2020). Once parous, their sensory systems become tuned to host-related odours, such as carbon dioxide and human skin volatiles, facilitating blood feeding for egg development (Omondi et al. 2019; Tallon et al. 2019). In the Queensland fruit fly (*Bactrocera tryoni*), olfactory responsiveness declines with age in both sexes. Male attraction to cue-lure drops sharply after 12 weeks, while female attraction to guava juice decreases gradually after six weeks due to reduced exploratory and orientation activity (Tasnin et al. 2020). Similarly, in *Drosophila melanogaster*, olfactory sensitivity and odorant receptor expression vary with age, with older flies showing weaker responses to food odours but stronger responses to pheromonal cues (Iliadi and Boulianne 2010). In the cotton bollworm (*Helicoverpa armigera*), pheromone responses are age-dependent: newly emerged females produce little pheromone, and males show low sensitivity, while both sexes reach peak responsiveness at sexual maturity (Chang et al. 2017; Liu et al. 2013). In the red flour beetle (*Tribolium castaneum*), both sexes respond to male-produced aggregation pheromones, but responses vary with concentration, strain, and age (Duehl et al. 2011). When insects emerge as adults, their olfactory and gustatory systems are often not fully mature. Sensitivity of olfactory receptor neurons (ORNs) increases as receptor, odorant-binding, and chemosensory proteins accumulate in sensory organs such as antennae and palps, enhancing odour detection (Guo and Smith 2022). In newly emerged insects, endocrine activity—particularly juvenile hormone (JH) and ecdysteroids—is low. These hormones regulate reproductive development, metabolism, and chemosensory sensitivity. In mosquitoes, low JH promotes sugar-seeking, whereas elevated JH and ecdysteroid levels after mating trigger host-seeking for blood meals (Tung and Fonseca 2024). Ageing also modifies neuromodulatory systems. Biogenic amines, including octopamine, dopamine, and serotonin, which influence olfactory processing and motivation, fluctuate with physiological state (Dacks et al. 2012). Consequently, olfactory centres such as the antennal lobe and mushroom body adjust their responsiveness to different odour types, including plant volatiles, pheromones, and host cues. In older insects, receptor neuron degeneration, reduced protein turnover, and weaker signal transduction lead to sensory decline and lower behavioural responses (DeVault et al. 2018). Thus, the combined effects of sensory maturation, hormonal regulation, and neuromodulation explain why insects’ olfactory responses are strongly dependent on physiological age, highlighting the need to consider age structure when deploying attractants or repellents in field-based pest management.

#### Reproductive status

Reproductive status greatly affects how insects detect and respond to odours. Hormonal and physiological changes linked to mating, egg development, or gestation modify both sensory sensitivity and behavioural priorities. Mating profoundly alters olfactory priorities in insects, reshaping how they respond to chemical cues relevant to reproduction and survival. In *Drosophila melanogaster* females, virgins are strongly attracted to male pheromones such as cis-vaccenyl acetate (cVA), but this attraction is suppressed after mating as seminal proteins reprogram females to focus on food and oviposition cues (Lebreton et al. 2014), making pheromone-based traps more effective for virgins than for mated individuals. Males show the opposite pattern, as mating increases their attraction to ammonia and protein-rich odours linked to oviposition substrates and enhances their dispersal and foraging activity (Wang et al. 2014), suggesting that food- or yeast-based attractants may better target mated males. Comparable shifts occur in mosquitoes, where olfactory sensitivity fluctuates throughout the gonotrophic cycle; after mating, females lose host attraction until ready for a blood meal and then reduce host-seeking again during egg development under hormonal and neuropeptide regulation (Attardo et al. 2003; Barredo and DeGennaro 2020; Duvall 2019). Similar patterns are observed in moths such as *Agrotis ipsilon* and *Spodoptera littoralis*, in which virgin males respond strongly to sex pheromones, whereas mated ones become more sensitive to host-plant volatiles through antennal lobe modulation (Barrozo et al. 2010; Kromann et al. 2015). These findings highlight that mating status determines which odours are most relevant to an insect’s behaviour, and incorporating this factor into semiochemical-based pest management can enhance trap performance and sustain control efficacy across reproductive stages.s.

#### Feeding condition

Nutritional state strongly influences insect olfactory behaviour. Starvation heightens sensitivity to food-related cues and suppresses aversive responses, improving resource-finding efficiency. In *Drosophila melanogaster*, this is mediated by neuromodulatory circuits involving short neuropeptide F (sNPF) and tachykinin that reconfigure olfactory processing (Ko et al. 2015). In blood-feeding triatomines, hunger enhances responses to CO₂ and host-related volatiles such as α-pinene and yeast odours, while reducing aversion to (−)-limonene and nonanal, thereby optimising host-seeking (Bodin et al. 2009; Reisenman et al. 2013). Starvation also increases antennal (EAG) responses at low to mid odorant concentrations, showing up to sevenfold stronger activity at night (Reisenman 2014). In female bed bugs (*Cimex lectularius*), starvation elevates host-seeking in unmated females but suppresses it in mated ones, indicating that nutritional and reproductive states jointly regulate olfactory sensitivity (Saveer et al. 2021). Mechanistically, starvation enhances presynaptic activity in olfactory sensory neurons via sNPF signalling, while insulin signalling downregulates sNPF receptor expression, jointly promoting food-search behaviour (Root et al. 2011; Slankster et al. 2020). Beyond starvation, other nutritional conditions also modulate odour detection by shifting sensory priorities toward needed resources. Protein deficiency stimulates olfactory-guided foraging for amino acid– and yeast-rich sources that support egg production (Liu et al. 2024; Toshima and Schleyer 2019). Low hemolymph sugar enhances activity in food-detecting neurons, whereas high sugar suppresses it, reducing food-seeking behaviour (Dus et al. 2015; May et al. 2019). In *Aedes albopictus*, sugar feeding delays host-seeking by upregulating fat body vitellogenin genes, particularly *Vg-2*, which suppresses attraction to host odours; this effect is reversed by *Vg-2* knockdown (Dittmer et al. 2019). Sugar deficiency impairs odour-guided learning and memory in *Venturia canescens*, reducing olfactory sensitivity and host-search efficiency by limiting neural and metabolic functions required for odour processing (Kishani Farahani et al. 2021). Similarly, diet lipid composition influences odour-guided behaviour in the Mediterranean fruit fly (*Ceratitis capitata*), where specific oil feeding altered attraction to odour cues and fatty acid profiles, suggesting that lipid deprivation can directly shape foraging and host-seeking behaviour (Rosa et al. 2018). Mineral and salt imbalances increase attraction to ion-rich sources, as seen in ants (Kaspari and Welti 2020). Poor larval nutrition can also reduce the size and sensitivity of adult olfactory neurons, altering odour-guided behaviour in *Manduca sexta* (Goyret et al. 2009). Taken together, these findings highlight that attractant or repellent efficacy in the field depends on the nutritional condition of the target insects, with starved individuals typically showing stronger responses.

#### Circadian rhythm

Circadian rhythms strongly shape olfactory sensitivity and behavioural responses in insects, ensuring that foraging, mating, and host-seeking occur when resources or mates are most accessible. Blowfly olfactory-driven oviposition is largely suppressed at night under natural conditions, but gravid females or unusually high nighttime temperatures can trigger activity, demonstrating that circadian rhythms gate odour detection and behaviour timing (Amendt et al. 2008). In mosquitoes, *Aedes aegypti* and *Anopheles gambiae* show peak attraction to host odours during their nocturnal activity phase, while responsiveness to sugar or plant volatiles is higher during daytime resting periods (Wijewardana et al. 2025). In *An. gambiae*, odorant-binding proteins (OBPs) and takeout proteins exhibit daily rhythmic expression, with antennal protein abundance and olfactory sensitivity peaking at pre-dusk and dusk, coinciding with increased host-seeking and blood-feeding behaviour (Rund et al. 2013). Circadian control also regulates cockroach olfactory-driven behaviours and food detection, with *Blattella germanica* feeding most actively between 19:00–22:00 and 04:00–05:00, *Periplaneta americana* during the early scotophase, and brown-banded cockroaches showing peak female calling and male responsiveness at night (Neupane 2022). In the nocturnal moth *Agrotis ipsilon*, olfactory-guided feeding peaks during the scotophase, indicating that attractant deployment is most effective when aligned with these nocturnal activity periods (Force et al. 2024). In *Spodoptera littoralis*, female sex pheromones synchronise and enhance male circadian activity, showing that olfactory cues can entrain male clocks and suggesting that pheromone-based attractants are most effective when deployed in phase with these socially entrained activity peaks (Ghosh et al. 2024). From a field perspective, circadian modulation means the efficacy of attractants or repellents is time-dependent, and deploying them outside peak activity periods may reduce effectiveness.

To mitigate the effects of physiological state on insect responses when deploying attractants or repellents in the field, several strategies can be adopted: (1) consider age structure by targeting physiologically mature adults that exhibit peak olfactory sensitivity and behavioural responsiveness, and by using age-specific lures—such as sugar-based attractants for newly emerged individuals and host- or oviposition-related cues for mature ones; (2) match attractant type to reproductive status by deploying pheromone traps primarily for virgin insects and food- or oviposition-based lures for mated individuals whose odour preferences have shifted; (3) account for nutritional condition by incorporating food-related cues when targeting starved populations, as hunger enhances olfactory responsiveness, and by avoiding prolonged exposure to attractants that may induce sensory adaptation; (4) synchronize deployment with circadian activity peaks to maximize response, for example by releasing host-seeking cues for nocturnal mosquitoes during evening hours or pheromone lures for moths at night when mating activity is highest; (5) adjust release rate and formulation to mimic natural odour emission patterns and maintain effective signal detection under varying metabolic or hormonal conditions; and (6) combine multimodal cues (olfactory, visual, or thermal) to strengthen attraction across individuals with differing physiological states, ensuring consistent detection and behavioural engagement under field conditions.

### Effect of Environmental Factors On Odour Cue Release, Dispersal, and Perception in Insects

**Fig. 7:**
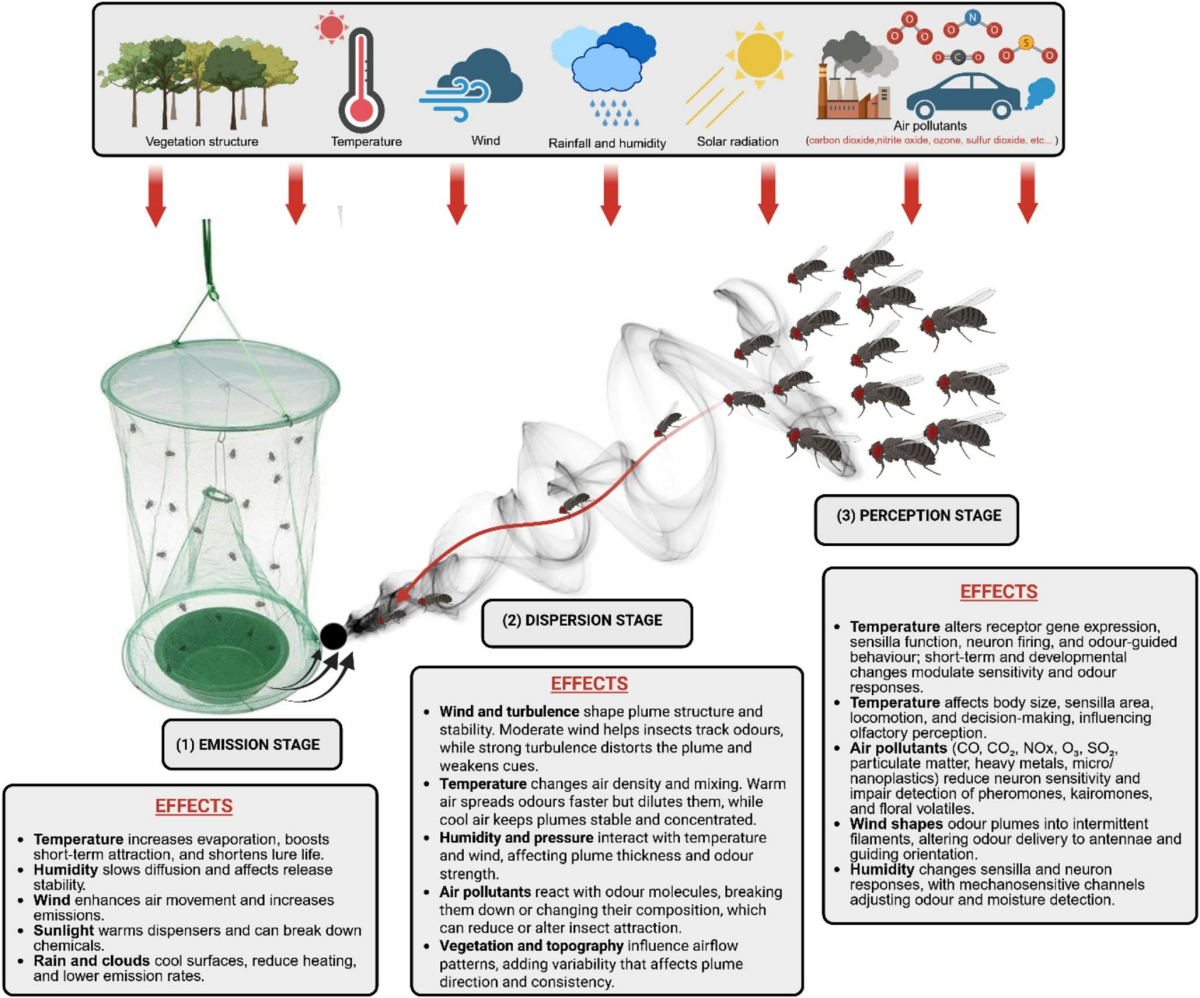
Illustration of how environmental factors influence chemical cues identified in the laboratory during their emission, dispersion, and perception stages in the field, affecting odour-guided responses in insects

Chemical cues identified in the laboratory, when deployed in the field, are influenced by environmental parameters at the emission stage (release from dispensers in traps), dispersion stage (odour plume formation and movement in the air), and perception stage (odour detection by olfactory receptors within antennal sensilla, leading to attraction or repulsion). The main environmental factors affecting odour cue performance across these stages include temperature, humidity, wind, solar radiation, rainfall, atmospheric pressure, air pollutants, vegetation structure, soil moisture, and topography (Fig. 7**)**.

#### Emission stage

Temperature is generally the most critical parameter governing odour emission from dispensers because it directly affects the physical performance of attractants and repellents. Higher temperatures increase the volatility of odour compounds, accelerating their evaporation. This enhances short-term dispersal but reduces cue persistence. Nielsen et al. (2019) showed that the release of methyl isonicotinate and related compounds from sachets, polyethylene bags, and cotton rolls followed zero-order kinetics, yet the rate increased sharply with temperature. At 35 °C, compounds volatilized much faster than at 15–25 °C, indicating that warmer conditions enhance immediate attraction but shorten lure longevity. Similarly, Torr et al. (1997) found that the emission of tsetse fly attractants—1-octen-3-ol, 4-methylphenol, and 3-n-propylphenol—from polyethylene sachets increased exponentially between 21 °C and 38 °C, with field release varying up to 100-fold depending on sunlight and temperature. van der Kraan and Ebbers (1990) reported that the release of tetradecen-1-ol acetates from polymeric formulations rose two- to two-and-a-half-fold as temperature increased from 15 °C to 25 °C. Likewise, Bradley et al. (1995) developed a predictive model showing that pheromone release from polyethylene dispensers scales reliably with ambient temperature. Together, these studies demonstrate that high temperatures accelerate evaporation, intensify short-term attraction, and reduce field longevity. Achieving stable release under fluctuating thermal conditions requires optimising dispenser design, material, and placement, as outcomes depend on compound chemistry, dispenser type, and airflow. Besides temperature, other environmental factors also influence volatility and emission rates. Relative humidity affects dispenser permeability and chemical stability, with high humidity often reducing diffusion. Wind speed influences the boundary layer around the dispenser, where stronger winds enhance mass transfer and increase release rates. Solar radiation can heat the dispenser or degrade volatiles through photolysis, indirectly promoting emission. Atmospheric pressure has a minor effect by slightly altering air density and diffusion. A good example of how these environmental factors interact is provided by Veršić Bratinčević et al. (2023), who found that the release rate of 2-phenethyl acetate, an attractant of the olive fruit fly (*Bactrocera oleae*), when loaded in the rubber septa dispenser, was negatively correlated with temperature, precipitation, cloud cover, and wind, but positively correlated with humidity and air pressure. Such environmental fluctuations can therefore destabilise release dynamics, alter compound ratios, and ultimately reduce the field efficiency of attractants or repellents loaded in dispensers.

#### Dispersion stage

During the dispersion stage, odour released from a dispenser forms a plume that interacts with the surrounding air. Its structure and behaviour are shaped by wind speed and direction, turbulence, temperature, humidity, solar radiation, pressure, topography, and vegetation. Among these, wind, turbulence, and temperature are the main factors determining plume shape, stability, and concentration. Houle and van Breugel (2023) showed that near-surface wind variability, which governs plume structure, changes with both wind speed and environmental complexity. Wind direction variability decreases as wind speed increases, but rises in heterogeneous landscapes such as forests and urban areas. They also found that turbulence intensity strongly correlates with fluctuations in wind direction, ranging from 15° to 75° over 1–10 min. This indicates that plume consistency depends on local turbulence and surface features. Insects, therefore, track odour plumes most effectively under intermediate wind speeds and moderate environmental complexity.

Lei et al. (2023) further demonstrated through computational fluid dynamics that turbulence introduces strong fluctuations and distortions in odour plumes, while wingbeat-induced airflow draws odorants closer to the antennae, enhancing detection. They also showed that odorant diffusivity, expressed by the Schmidt number, influences plume consistency and odour detectability, highlighting that fine-scale wind dynamics directly affect olfactory tracking. Once released, temperature modifies plume behaviour by altering air density and turbulence. At higher temperatures, air becomes less dense and more turbulent, enhancing dispersion but causing faster dilution and weaker cues over distance (Conchou et al. 2019; Riffell et al. 2008). Warm air also promotes vertical mixing, which destabilises the plume and hinders source tracking (Justus et al. 2002; Murlis et al. 1992, 2000). In contrast, cooler air promotes stable layers that maintain concentrated odour filaments detectable over longer periods (Cardé and Willis 2008). Temperature also affects plume chemistry. Volatile compounds degrade or oxidise more rapidly under warm conditions, modifying odour blends and potentially altering insect responses (Pinto-Zevallos and Blande 2024). These effects, combined with adverse weather interactions among humidity, wind speed, temperature, and barometric pressure, can disrupt odour-guided foraging in insects such as *Cotesia glomerata* (Vosteen et al. 2020).

Beyond these meteorological factors, air pollutants from human activity can alter odour plume formation, composition, and movement by changing the chemical and physical properties of air. Pollutants such as ozone (O₃), nitrogen oxides (NOₓ), sulfur dioxide (SO₂), and particulate matter react with volatile organic compounds (VOCs) released from dispensers, breaking down or transforming key odorants before they reach an insect’s antennae (Knaden et al. 2022; Fuentes et al. 2016). Oxidants, such as ozone, rapidly degrade terpenes, aldehydes, and esters, thereby shortening their atmospheric lifetimes and distorting the original odour blend (McFrederick et al. 2008). These reactions can eliminate critical components of attractant mixtures or create new compounds that are irrelevant or even repellent to insects (Venkateswaran et al. 2023). In nocturnal hawkmoths, Chan et al. (2024) showed that nitrate radicals (NO₃) degrade floral scent compounds (β-pinene and *cis*-β-ocimene), reducing the insects’ ability to detect host odours and leading to fewer pollination visits. Exposure of floral volatiles to NOx degraded α-farnesene and α-terpinene while increasing the abundances of α-pinene, 3-carene, and ρ-cymene, thereby altering the blend composition and significantly reducing odour recognition in honeybees (*Apis mellifera*) (Girling et al. 2013). Air pollutants also alter boundary-layer dynamics by increasing aerosol loading. Higher particulate concentrations enhance light scattering and reduce thermal gradients, thereby dampening turbulence and slowing vertical mixing. Some pollutants act as condensation nuclei, modifying humidity and affecting volatile release rates. Pollutant-induced temperature inversions can trap odorants near the ground or block upward diffusion, altering the spatial distribution of the plume (Zhang et al. 2025).

Vegetation and topography further shape airflow, plume meandering, and local accumulation of volatiles. Vegetation modifies both chemical and physical plume structure. Plant architecture, surface adsorption, and background volatiles alter odour composition and turbulence, affecting how insects perceive these cues. Topographic features such as valleys or basins can locally concentrate volatiles and change the odour spatial structure before reaching the insect antennae. These interactions are well captured by the “odorscape” concept (Conchou et al. 2019). Together, vegetation and topography determine how odour molecules are delivered to sensory organs, shaping receptor neuron input and influencing olfactory perception. In insect pest management, these effects imply that vegetation density, canopy structure, and terrain can alter the efficiency of attractants and repellents deployed in the field. Dense vegetation may trap or adsorb volatiles, weakening signal strength and limiting plume spread, while background plant odours can mask synthetic cues and reduce insect responsiveness. Complex topography can fragment or redirect plumes, making it harder for insects to locate the source. Conversely, valleys or basins may enhance odour accumulation but restrict cue dispersal to localised areas.

Collectively, these processes make odour plumes more fragmented, chemically altered, and unpredictable, reducing the ability of target insects to detect the odour and thereby lowering the effectiveness of attractants or repellents deployed in the field.

#### Perception stage

Here, odorant molecules from the odour plume enter the antennal sensilla, bind to specific olfactory receptors, and trigger neural signals that the insect brain interprets to drive attraction or avoidance. Temperature strongly influences this process by affecting biochemical and physiological functions in insects (Colinet et al. 2015). In *Drosophila melanogaster*, shifts from 21 °C to 30 °C altered 95 heat- and olfaction-related genes, including *orco*, leading to sensilla-specific adjustments that maintain odour detection under heat stress (Riveron et al. 2013). A similar pattern was observed in *Apis cerana cerana*, where the mRNA and protein levels of *AcerOr1* and *AcerOr2* peaked at 25 °C, suggesting temperature-dependent regulation of olfactory genes (Guo et al. 2025). At the neuronal level, temperature modifies the firing dynamics of olfactory receptor neurons (ORNs), with heat reducing and cold enhancing olfactory sensitivity in *D. melanogaster*, reflecting short-term acclimation to changing odorant volatility (Riveron et al. 2009). Martin et al. (2011) further demonstrated that antennal sensitivity increases with temperature but decreases under cold conditions, confirming that ORN activity is dependent on thermal variation. In *Spodoptera littoralis*, developmental temperature interacts with body size to shape antennal sensitivity, as males reared at 33 ± 5 °C and 25 ± 5 °C had comparable mean sensitivities, yet body mass and antennal response correlated positively only at higher temperatures (Bagni et al. 2024), implying that temperature may influence olfaction indirectly by altering body size or sensilla surface area. In mosquitoes, Lahondère et al. (2023) found that *Aedes aegypti* antennae produced the strongest electroantennogram responses at 25 °C, indicating an optimal thermal range for olfactory performance. Temperature also shapes odour-guided behaviour by modulating locomotor activity, motivation, and decision-making. In three drosophilid species (*D. novamexicana*, *D. virilis*, and *D. ezoana*), Baleba et al. (2023) reported that developmental (18 °C, 25 °C) and experimental (10–35 °C) temperatures altered responses to methyl salicylate, guaiacol, and isopropyl benzoate, with odour valence shifting according to species’ thermal adaptation. Likewise, *Ae. aegypti* displayed stronger attraction to CO₂ at 30 °C than at 20–25 °C, suggesting that warmer conditions enhance host-seeking behaviour (Lahondère et al. 2023). Together, these findings demonstrate that temperature affects insect olfactory systems at molecular, neuronal, and behavioural levels, thereby shaping their ability to perceive attractants or repellents in the field.

In addition to temperature, the detection of odour molecules that have reached the olfactory sensory neurons of the targeted insects can be influenced by prior exposure to primary and secondary atmospheric pollutants such as carbon monoxide (CO), carbon dioxide (CO₂), cadmium(Cd), nitrogen oxides (NOx), ozone (O₃), sulfur dioxide (SO₂), and particulate matter (PM₂.₅/PM₁₀), as well as associated heavy metals (HMs) and micro- and nanoplastics (MPs/NPs) (Pinto-Zevallos et al. 2025). Several studies have shown that air pollutant exposure alters the insect olfactory system by reducing the sensitivity of olfactory sensory neurons, thereby impairing behavioural responses to odour stimuli. For example, Wang et al. (2023) demonstrated that short-term exposure to particulate matter severely compromises olfactory perception in the housefly (*M. domestica*), where particulate accumulation on the antennal surface increased with pollution severity, leading to reduced antennal responses to reproductive and food odours. In the pine sawyer beetle (*Monochamus galloprovincialis*), Álvarez et al. (2015) showed that smoke exposure alters pheromone and kairomone detection by activating or inhibiting specific olfactory receptor neurons, disrupting mate and host location. Comparable effects of pollutants have also been reported in beneficial insects such as pollinators and parasitoids. In the western honey bee (*Apis mellifera*), exposure to ozone affects antennal responses to (Z)−3-hexenyl acetate (Dötterl et al. 2016). Vanderplanck et al. (2021) found that high ozone (O₃) concentrations impair odour detection and behavioural responses in the bumblebee (*Bombus terrestris*) and the fig wasp (*Blastophaga psenes*), reducing their ability to perceive and respond to floral volatiles. In the fire ant (*Solenopsis invicta*), Xiao et al. (2024) reported that cadmium exposure suppresses expression of the odorant-binding protein SiOBP14, disrupts odorant receptor neuron signalling, and weakens olfactory-driven behaviours. These findings highlight that atmospheric pollutants can directly disrupt insect olfactory function, reducing their ability to detect vital chemical cues. In the context of insect pest management, this implies that the efficiency of odour-based control strategies—such as pheromone traps, attractant lures, or repellent formulations—may decline under polluted field conditions, as target insects might not properly perceive or respond to the deployed semiochemicals, leading to reduced trap catches and weakened behavioural manipulation. Also, wind and humidity directly influence how insects perceive odours from an odour plume once the molecules reach their sensory organs. Wind determines how odorant molecules arrive at the antenna, shaping the temporal pattern of stimuli insects receive. Murlis et al. (1992) showed that turbulent airflow breaks odour plumes into intermittent filaments, leading insects to perceive odours as brief, variable pulses that guide orientation toward the source. Vickers (2000) found that insects use these short odour bursts, together with wind direction, to adjust flight and maintain contact with the plume when the signal fades. Rigolli et al. (2022) further demonstrated that under strong turbulence, odour signals become fragmented and diluted, forcing insects to interpret irregular pulses rather than continuous gradients. At the neuronal level, Tuckman et al. (2022) reported that changing wind patterns not only shape the external odour field but also influence how olfactory information is encoded and perceived within the insect’s nervous system. Similarly, humidity affects the sensillar environment and receptor neuron responsiveness, as shown in *Drosophila*, where the mechanosensitive channel TMEM63 mediates humidity sensing in olfactory sensilla, modifying neural responses to odour and moisture stimuli (Li et al. 2022). These findings imply that the effectiveness of attractants or repellents in the field also depends on local wind and humidity conditions.

Taken together, to improve odour-based pest control tools under fluctuating environmental conditions, mitigation should target the three critical stages of odour cue performance—release, dispersal, and perception. (1) **Emission stage**: Use temperature-stable formulations such as polymeric matrices, wax coatings, or microcapsules to regulate evaporation across thermal gradients, and select dispenser materials like low- or high-density polyethylene based on compound volatility. Include stabilising agents such as antioxidants or UV absorbers to prevent degradation by sunlight and ozone, and place traps in shaded or sheltered locations to reduce thermal stress and excessive wind-driven release. Humidity effects can be managed with low-permeability or coated dispensers, and elevating traps prevents wash-off from rain or high soil moisture. Dispenser placement should also consider local vegetation and topography to maintain consistent release. (2) **Dispersion stage**: Position traps to account for wind direction and landscape heterogeneity, optimising odour plume structure and reach. Physical features such as vegetation strips or semi-permeable barriers can stabilise airflow, and lure replacement should coincide with moderate wind, humidity, and temperature conditions that favour odour tracking. Multi-component blends, such as host odour–CO₂ combinations, improve robustness when individual volatiles degrade. Semi-sealed dispensers and scavengers such as ascorbic acid or phenolic compounds can limit exposure to ozone and nitrogen oxides, while resistant odorants like alcohols or ketones can replace more reactive terpenes in polluted areas. (3) **Perception stage**: Deploy traps during thermally optimal periods for target insects, and integrate olfactory cues with visual or thermal signals to compensate for reduced sensitivity under extreme conditions or pollution. Ensure insects can access and track the plume without obstruction from dense vegetation or complex terrain, and align deployment with intermediate wind speeds, optimal humidity, and clean air to maximise olfactory detection. Together, these approaches enhance the stability, reliability, and effectiveness of odour-based control tools despite environmental variability.

## General Conclusion and Future Prospects

The management of filth flies remains a pressing challenge for global public health and livestock economies. This review synthesises major advances in decoding the semiochemical language of these pests, revealing a rich set of kairomones, pheromones, and repellents that shape their key behaviours. From host-seeking attractants like 1-octen-3-ol and dimethyl trisulfide to oviposition stimulants and the sex pheromone (Z)−9-tricosene, research has established a strong chemical toolkit with proven potential for monitoring and control.

Yet, translating these chemical discoveries into field-effective applications remains complex. The efficacy of any semiochemical depends on three interacting factors: the fly’s internal physiology (age, nutritional and reproductive state), its behavioural flexibility (learning and habituation), and environmental conditions that affect odour release, plume structure, and neuronal perception. A potent laboratory lure may fail in the field if it mismatches the motivational state of the target fly or if environmental factors such as wind, heat, or pollutants distort the chemical signal.

The future of odour-based management demands a more integrated, second-generation approach that combines innovation with ecological realism. This will require:

1. **Answering foundational questions**: Prioritising studies on host-specific odour signatures, microbial symbionts, and the molecular basis of olfactory detection provides the foundation for designing precise interventions.
2. **Developing intelligent**,** multi-modal strategies**: Moving beyond single compounds toward synergistic blends that act on multiple behaviours and sensory pathways to reduce habituation and improve trap performance.
3. **Engineering resilient technologies**: Advancing formulation science to produce affordable, climate-tolerant dispensers that deliver stable, long-lasting odour release.
4. **Embracing a One Health framework**: Semiochemical tools can reduce filth fly burdens without the ecological and resistance risks of insecticides, supporting animal welfare, ecosystem health, and pathogen control.
5. **Fostering translational collaboration**: Bridging basic chemical ecology with applied pest management through partnerships among entomologists, chemists, engineers, and industry to produce practical, scalable solutions.

While the odour-mediated world of filth flies is complex, it remains their greatest vulnerability. By deepening our understanding of their chemical ecology and addressing the challenges identified here, we can shift from observing their behaviour to manipulating it. This shift will drive the development of a new generation of sustainable, effective, and field-ready tools for integrated management of filth flies.

## Data Availability

No datasets were generated or analysed during the current study.
